# Spontaneous Neuronal Activity in Developing Neocortical Networks: From Single Cells to Large-Scale Interactions

**DOI:** 10.3389/fncir.2016.00040

**Published:** 2016-05-24

**Authors:** Heiko J. Luhmann, Anne Sinning, Jenq-Wei Yang, Vicente Reyes-Puerta, Maik C. Stüttgen, Sergei Kirischuk, Werner Kilb

**Affiliations:** ^1^Institute of Physiology, University Medical Center of the Johannes Gutenberg University MainzMainz, Germany; ^2^Institute of Pathophysiology, University Medical Center of the Johannes Gutenberg University MainzMainz, Germany

**Keywords:** development, cerebral cortex, subplate, spontaneous activity, somatosensory cortex, columnar organization, rodent, human

## Abstract

Neuronal activity has been shown to be essential for the proper formation of neuronal circuits, affecting developmental processes like neurogenesis, migration, programmed cell death, cellular differentiation, formation of local and long-range axonal connections, synaptic plasticity or myelination. Accordingly, neocortical areas reveal distinct spontaneous and sensory-driven neuronal activity patterns already at early phases of development. At embryonic stages, when immature neurons start to develop voltage-dependent channels, spontaneous activity is highly synchronized within small neuronal networks and governed by electrical synaptic transmission. Subsequently, spontaneous activity patterns become more complex, involve larger networks and propagate over several neocortical areas. The developmental shift from local to large-scale network activity is accompanied by a gradual shift from electrical to chemical synaptic transmission with an initial excitatory action of chloride-gated channels activated by GABA, glycine and taurine. Transient neuronal populations in the subplate (SP) support temporary circuits that play an important role in tuning early neocortical activity and the formation of mature neuronal networks. Thus, early spontaneous activity patterns control the formation of developing networks in sensory cortices, and disturbances of these activity patterns may lead to long-lasting neuronal deficits.

## Introduction

Neuronal populations have the ability to self-organize into networks that promote the generation of spontaneous, correlated neuronal activity already at earliest developmental stages. Isolated cortical neurons in dissociated cultures generate after a few days *in vitro* spontaneous action potentials and intracellular calcium transients at irregular intervals (Ramakers et al., 1990; Opitz et al., 2002; Sun et al., 2010). With the developmental shift from electrical to chemical synaptic transmission and increasing axonal connectivity, developing neuronal cultures generate repetitive burst discharges, which are then synchronized over large fractions of the culture. At this developmental stage, spontaneous activity is organized in repetitive spike patterns, with a subgroup of neocortical neurons exhibiting a high degree of synaptic inputs and outputs (“hub” neurons; Sun et al., 2010). A similar maturation of spontaneous burst activity can be also observed in organotypic neocortical slice cultures (Gorba et al., 1999; Baker et al., 2006). Intriguingly, these activity patterns generated autonomously by the self-organization of networks from isolated cortical neurons resemble many basic properties of spontaneous network activity observed in the immature neocortex *in vivo*.

*In vivo*, the early postnatal development of spontaneous activity in sensory neocortical areas has been studied in various mammalian species, including mice, rats, ferrets, monkeys and humans. The patterns of spontaneous synchronized network activity in these different species show surprising similarities when the developmental status of the neocortical network is taken into account (for review see Khazipov and Luhmann, 2006). Since rodents offer the advantage of a rich repertoire of experimental manipulations and read-outs, experimental studies in these species provided a better understanding of the early development of physiological and pathophysiological properties of the cerebral cortex in humans. At the same time, these studies offer the opportunity to obtain evidence for the causal relationship between network activity and the structural and functional development of the cerebral cortex.

Both *in vitro* and *in vivo* results strongly suggest that spontaneous synchronized burst activity represents a functional hallmark of developing neocortical networks. In addition, theoretical considerations, experimental evidence and clinical findings suggest that such spontaneous, correlated activity is fundamental for the functional maturation of sensory cortices (Thivierge, 2009; Ben-Ari and Spitzer, 2010; Kirkby et al., 2013; Rahkonen et al., 2013; Levin, 2014). This review aims to provide an update on the properties of spontaneous activity patterns in sensory neocortical areas during early stages of development and how these patterns are generated. Subsequently, we will discuss the physiological relevance of these early activities and how pathophysiological disturbances in spontaneous activity may alter the maturation of cortical networks. Since the developing cerebral cortex shows prominent anatomical and physiological changes during late prenatal and early postnatal stages, we will first briefly summarize the structure of the developing cerebral cortex.

## The Structure of the Developing Cerebral Cortex

**Figure 1:**
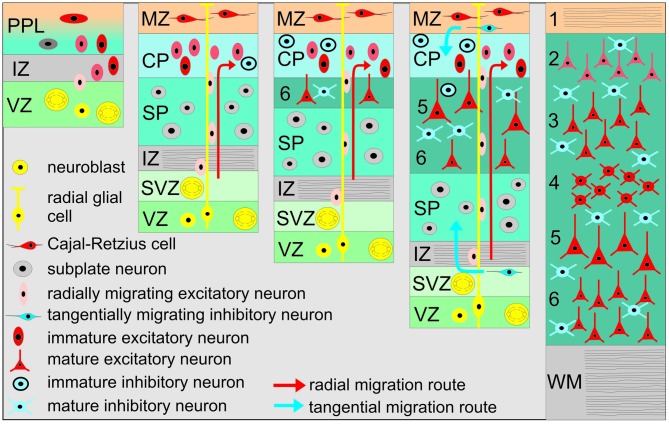
**Schematic diagram illustrating the basic principles of neocortical development.** The earliest cohort of generated neurons forms the primordial plexiform layer (PPL), which includes Cajal-Retzius (CR) and subplate neurons (SPNs). Later generated neurons migrate along the processes of radial glial cells and split the PPL into the superficial marginal zone (MZ) and the SP. Later born neurons migrate toward the pial surface and detach from radial glial processes at the border of the MZ into the cortical plate (CP), thus establishing the inside first—outside last orientation of the neocortex. IZ, Intermediate zone; VZ, Ventricular zone; SVZ, Subventricular zone; WM, White matter. Neocortical layers are numbered by 1–6.

Although the overall structural and functional development of the cerebral cortex during early stages is similar in different mammalian species ranging from mouse to human, the time points and periods of distinct developmental processes (e.g., neurogenesis, migration, differentiation, synaptogenesis, apoptosis) differ due to large differences in gestation periods (for review see Molnár et al., 2006; Molnár and Clowry, 2012). In most species the six-layered cerebral cortex is generated during prenatal and early postnatal stages following an inside first—outside last pattern. Thus, neurons in layer (L) 6 are born first in the ventricular zone (VZ) and migrate to the pial surface to split the primordial plexiform layer (PPL) into the superficial marginal zone (MZ) and the profound subplate (SP; Figure 1). Neurons in L3 and L2 are generated later and migrate through the lower layers, which are populated with postmigratory neurons that display more mature properties. Two populations of very early generated and transient neurons fulfill important roles in corticogenesis (for review see Luhmann, 2013): (1) Cajal-Retzius neurons (CRNs) are located in the MZ (which later becomes L1), and control radial neuronal migration (for review see Kirischuk et al., 2014); and (2) subplate neurons (SPNs) are located between the White matter (WM) and L6, playing important roles in early thalamocortical circuits and the maturation of the neocortical architecture (for review see Kanold and Luhmann, 2010). CRNs as well as SPNs are among the earliest generated forebrain neurons and show relatively mature functional properties in the newborn rodent cortex, such as repetitive action potential discharges and prominent synaptic inputs. It has been suggested that highly connected SPNs may act as amplifiers and hub neurons in early neocortical networks (Luhmann et al., 2009; Kanold and Luhmann, 2010). When all neocortical layers have been generated (in rodents at postnatal day [P] 4–5, in full-term human infants shortly before birth), most CRNs and a substantial fraction of SPNs disappear and the developing neocortical networks undergo extensive experience-dependent reorganization during the subsequent critical period for the different sensory systems. While virtually all CRN have been shown to perish by apoptosis (Chowdhury et al., 2010), the fate of SPN is a matter of discussion (Marx et al., 2015).

## Spontaneous Neocortical Activity at Prenatal and Early Postnatal Stages

**Figure 2:**
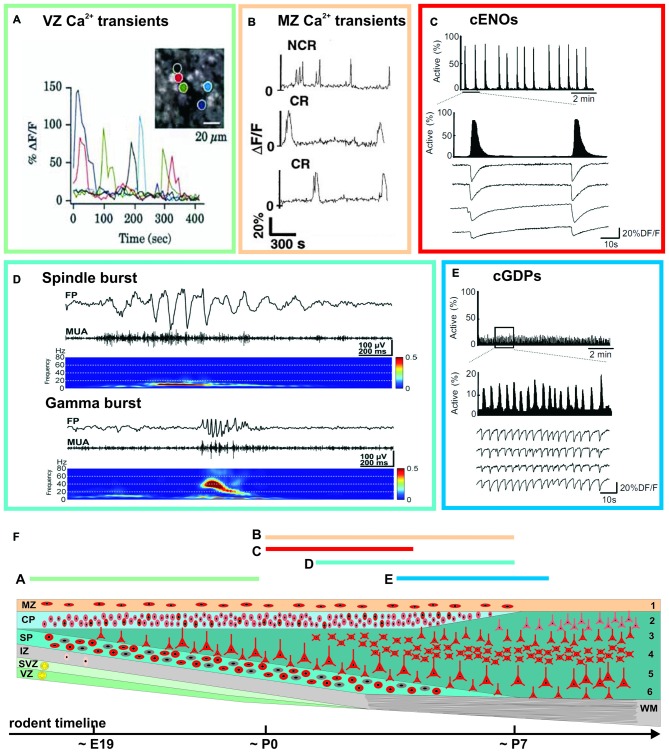
**Examples of spontaneous activity patterns at specific early ontogenetic stages (A–E) and schematic illustration of the developmental trajectory (F).** Rodent timescale included below based on Ignacio et al. (1995). The approximate occurrence of these events is indicated by the color-coded bars and corresponding letters. **(A)** Correlated and uncorrelated slow calcium transients occurring in the VZ of a E15 rat cortex (modified from Owens et al., 2000). **(B)** Spontaneous calcium transients of CR and non-Cajal-Retzius (NCR) neurons in the MZ of a postnatal rat neocortex (modified from Schwartz et al., 1998). **(C)** Calcium imaging reveals spontaneous cortical early network oscillations (cENOs) in P3 rat neocortical neurons. **(D)** Both spindle and gamma bursts occurring spontaneously in the somatosensory cortex (S1) of a P3 rat (modified from Yang et al., 2009). **(E)** In P6 rat neocortical slices, cENOs are replaced by cortical giant depolarizing potentials (cGDPs; **C** and **E** modified from Allène et al., 2008). See main text for details.

Prominent spontaneous activity can be observed in the neocortex at surprisingly early developmental stages, both at the single cell level as well as at the network level (Figure 2). Spontaneous calcium transients have been reported in mouse neocortical slices already at embryonic day (E) 16 (Corlew et al., 2004), i.e., 4–5 days before natural birth of the mouse and at a time point when the six neocortical layers have not even been formed. This low frequency (<1 min^−1^) activity is correlated between a large set of neurons, is sensitive to tetrodotoxin (TTX, a blocker of voltage-gated sodium channels), and relies on voltage-gated calcium channels, indicating that electrical activity with subsequent calcium influx is essential for its occurrence (Corlew et al., 2004). At this developmental stage spontaneous calcium transients can also be observed in the proliferative epithelium of the VZ (Figure 2A). These calcium transients are independent of electrical activity, glutamate or GABA receptors and require calcium release from intracellular stores (Owens et al., 2000). They are most probably representing spatially restricted, slowly propagating calcium waves, which are mediated by connexin hemichannels and purinoceptors (Weissman et al., 2004).

Already at the first postnatal day (P0), a repertoire of large scale network events appear in the immature neocortex. The so-called cortical early network oscillations (cENOs) have been found in neocortical slice preparations of newborn rats (Figure 2C). These spontaneous and TTX-sensitive cENOs usually start in the posterior cerebral cortex, occur approximately every 2 min and propagate with ~2 mm/s over the whole cortex to the anterior pole (Garaschuk et al., 2000). At all ages cENOs are completely and reversibly blocked by AMPA and NMDA receptor antagonists. Using large acute brain slice preparations Namiki et al. (2013) demonstrated that a comparable activity pattern in P1–P6 rats is triggered by a population of L3 neurons, that are autonomously active. Spontaneous calcium waves (reflecting the correlated activity of a few thousand cells, and resembling the *in vitro* cENOs) have been also observed *in vivo* under non-anesthetized conditions (Adelsberger et al., 2005).

Using calcium imaging in combination with single-cell and field potential recordings, Allène et al. (2008) demonstrated by using somatosensory neocortical slices from E20 to P9 rats that cENOs are developmentally followed by another distinct spontaneous activity pattern, the so-called cortical giant depolarizing potentials (cGDPs, Figure 2E). They appear around P4–P5 and differ from cENOs by: (1) their higher occurrence (~8 min^−1^); (2) their substantially faster kinetics; and (3) they are only partially affected by AMPA/NMDA receptor antagonists, but depend mainly on depolarizing GABA_A_ receptor-mediated transmission (Allène et al., 2008). GDPs have been initially observed and extensively studied in the hippocampus of newborn rodents (Ben-Ari et al., 1989), and later also in hippocampal slices from fetal monkeys (Khazipov et al., 2001; for review see Ben-Ari, 2014). As a conclusion it has been postulated that, in the neocortex, spontaneous cGDPs synchronize localized neuronal assemblies.

At a later developmental stage, at around P10–P11, so-called slow activity transients (SATs) have been recorded in the visual cortex of non-anesthetized rats before eye opening (Colonnese and Khazipov, 2010). SATs are long (~10 s) and large [>1 mV in local field potential (LFP) recording] spontaneous events produced by the summation of rapid oscillatory bursts (15–30 Hz). In the cerebral cortex SATs spread horizontally and locally synchronize network activity via the rapid oscillations. SATs have been also recorded with direct current (DC) coupled EEG recordings from sleeping preterm human babies (Vanhatalo et al., 2005). These SATs, which in humans are mostly confined to the prenatal stage, are slow and large (up to 0.8 mV) voltage deflections which nest oscillatory activity up to 30 Hz (Tolonen et al., 2007).

Whereas the events described so far mainly cover activity patterns which propagate over large-scales, *in vivo* electrophysiological recordings from rodent cortex revealed local and distinct spontaneous activity patterns synchronizing only confined neuronal networks (Figure 2D). A typical example of such local spontaneous activity in the newborn (~P3) rodent cerebral cortex are gamma oscillations, which appear spontaneously every 10–30 s, have a duration of 100–300 ms and a frequency of 30–40 Hz (Yang et al., 2009; Minlebaev et al., 2011; for review see Khazipov et al., 2013). Gamma oscillations are restricted to local functional columns. Inhibitory synaptic transmission plays only a minor role in their generation, suggesting that they are functionally distinct from the typical gamma oscillation observed in mature neocortex (Khazipov et al., 2013). A second typical pattern is the spindle bursts, i.e., local and short network oscillations in a frequency range of 10–20 Hz (Figure 2D), which have been recorded in visual and somatosensory cortical areas of newborn rats (Khazipov et al., 2004; Hanganu et al., 2006; Minlebaev et al., 2007; Yang et al., 2009, 2013; for review see Yang et al., 2016). They occur spontaneously every ~10 s and have a duration of 0.5–3 s.

In addition to these activity patterns described mainly in the cortical plate (CP) and/or cortical layers 2–6, correlated network activity has been also found in the MZ of rodent perinatal neocortex (Figure 2B). In this layer spontaneous calcium transients occur at a low frequency (<0.5 min^−1^) and are uncorrelated, but yet reveal an underlying network of connected neurons (Schwartz et al., 1998). The correlation between individual neurons is abolished in the presence of TTX and by inhibition of AMPA, NMDA or GABA-A receptors (Aguiló et al., 1999).

In summary, electrophysiological and imaging studies in different neocortical areas of various mammalian species have revealed a rich repertoire of spontaneous activity patterns, which are present during distinct phases of late prenatal and early postnatal development (Figure 2F). Notably, these patterns mostly develop from repetitive activity (cENOs and cGDPs) to more complex activity motives. In the next paragraphs, we will review our current understanding on the cellular elements and the mechanisms underlying these neocortical activity patterns.

## Cellular Elements Underlying Spontaneous Neocortical Activity at Prenatal and Early Postnatal Stages

A number of reports identified specific neuronal populations that are suited to generate these spatially and temporally distinct activity patterns in the developing neocortex. Spontaneous calcium transients in the proliferative epithelium of the VZ shown in acute cortical preparations from embryonic rats and mice are probably generated in proliferating radial glial cells (Owens et al., 2000; Weissman et al., 2004). Already at E16 some neurons in the mouse neocortex show high-frequency repetitive action potential discharges and spontaneous glutamatergic and GABAergic synaptic inputs (see Figure 4 in Kilb et al., 2011). Such early pioneer populations may underlie the occurrence of TTX-sensitive activity transients in the embryonic neocortex (Corlew et al., 2004).

The cellular mechanisms underlying the activation of the MZ has been studied in tangential slice and whole-hemisphere preparations of newborn rat cerebral cortex using optical imaging and patch-clamp recordings. These experiments showed that the major neuronal class involved in these activity transients are CRNs (Schwartz et al., 1998; Aguiló et al., 1999). Pharmacological experiments revealed that the correlation between individual CRNs observed during spontaneous calcium transients is abolished in the presence of TTX and by inhibition of AMPA, NMDA or GABA-A receptors, indicating their dependence on synaptic transmission (Aguiló et al., 1999). On the other hand, the frequency of these spontaneous calcium transients is unaffected by TTX or inhibition of GABA and glutamate receptors (Schwartz et al., 1998; Aguiló et al., 1999), suggesting that additional mechanisms drive these events. One possible candidate is the nonsynaptically released neurotransmitter taurine, which has been shown to mediate the propagation of excitation in the MZ via glycine receptors (Qian et al., 2014) and which excites CRNs (Kilb et al., 2002). A similar role of taurine has been described in the CP, where taurine selectively excites GABAergic interneurons in the CP, which enhance network excitability by excitatory GABAergic postsynaptic potentials (Sava et al., 2014). However, as the synaptic targets of neocortical CRNs have not been functionally identified yet (Kirischuk et al., 2014), the role of CRNs in the generation or transmission of spontaneous activity remains unclear.

Another transient cell population which drives the activity in the developing neocortical network are SPNs. It is well documented in different species and in various sensory neocortical areas that during early development SPNs receive a strong thalamocortical synaptic input mediated by AMPA and NMDA receptors (Friauf et al., 1990; Friauf and Shatz, 1991; Hanganu et al., 2001, 2002; Hirsch and Luhmann, 2008; Zhao et al., 2009). Furthermore, SPNs receive different neuromodulatory (e.g., cholinergic) inputs and are well integrated in a dense network of intracortical connections (for review see Kanold and Luhmann, 2010). Activation of nicotinic receptors (Hanganu and Luhmann, 2004), muscarinic receptors of predominantly m1/m5-subtype (Hanganu et al., 2009), glycine receptors (Kilb et al., 2008) and GABA-A receptors (Hanganu et al., 2001, 2002) causes an excitation of SPNs, which may elicit a local and transient network oscillation in a frequency range of 10–20 Hz (Dupont et al., 2006; Hanganu et al., 2009). This activity pattern, related to spindle bursts at the network level, depends on an intact functional SP (Dupont et al., 2006; Yang et al., 2009; Tolner et al., 2012). Since SPNs neurons are capable to intrinsically discharge at 10–20 Hz when activated by cholinergic mechanisms (Hanganu et al., 2009) and are electrically coupled in a columnar manner to neighboring and developing CP neurons via neuronal, connexin-36 containing gap junctions (Dupont et al., 2006), it has been suggested that SPNs act as local amplifiers of this early cortical activity (for review see Luhmann et al., 2009). The interplay between phasic and tonic synaptic activation elicits a 10–20 Hz oscillatory response in SPNs, which synchronizes the activity of a local, columnar network, thereby producing spindle bursts.

## Generation of Spontaneous Neocortical Activity—More Than One Circuit and One Brain Region!

The question how and where early neocortical activity patterns are generated is still debated. Clearly, the mechanisms underlying network activity critically depend on the developmental stage, and in rodents, which show a fast development during the perinatal phase, the mechanisms of activity generation change within 2–3 days (for review see Allene and Cossart, 2010; Kilb et al., 2011). Synchronized network activity may either be triggered by a specific brain region, circuit or a discrete subset of pacemaker neurons, or may emerge during early development as an intrinsic property of the network.

Pacemaker properties within cortical networks have been identified in slice cultures of the mouse neocortex, which reveal synchronized spontaneous activity propagating as a wave from a ventrolateral pacemaker region (Lischalk et al., 2009). However, activity originating from this ventrolateral region is itself often triggered by preceding activity in the septal nuclei (Conhaim et al., 2010). These spontaneous waves occur in organotypic slice cultures between E18 and P12 and show prominent developmental changes in their transmitter dependence and propagation patterns (Conhaim et al., 2011). Both *in vitro* and *in vivo* studies have shown that synchronized spontaneous activity in the neocortex during the late embryonic and early postnatal phase often depends on gap junctional coupling (Yuste et al., 1995; Kandler and Katz, 1998; Owens and Kriegstein, 1998; Sun and Luhmann, 2007; Yang et al., 2009) with a contribution of neuronal, connexin-36 containing electrical synapses (Dupont et al., 2006; Wagner and Luhmann, 2006; Hanganu et al., 2009; for review see Uhlén et al., 2015). A role of gap junctions, including connexin-36, has also been demonstrated recently in the generation of spontaneous depolarizations in human fetal cortex during the second trimester of gestation (Moore et al., 2014). Computational studies have shown that gap junction-coupled networks can produce a wide range of spontaneous activity patterns (Kepler et al., 1990; Sherman and Rinzel, 1992; Bennett and Zukin, 2004; Tseng et al., 2008; Uhlén et al., 2015). However, for the interpretation of experimental studies using gap junction blockers it should be noted that some compounds are not very specific and have a number of side effects, such as inhibiting NMDA receptors (Chepkova et al., 2008) or voltage-gated calcium channels (Vessey et al., 2004).

It remains to be studied in more detail whether developing neocortical neurons possess intrinsic pacemaker properties to generate and drive distinct synchronized activity patterns (Luhmann et al., 2003; Sun et al., 2012). Hyperpolarization-activated cyclic nucleotide-gated (HCN) channels constituting the molecular substrate of hyperpolarization-activated current (*I*_h_) could potentially fulfil an important functional role (for review see Bender and Baram, 2008), as e.g., clearly demonstrated for thalamic relay neurons expressing HCN channels (for review see Pape, 1996).

**Figure 3:**
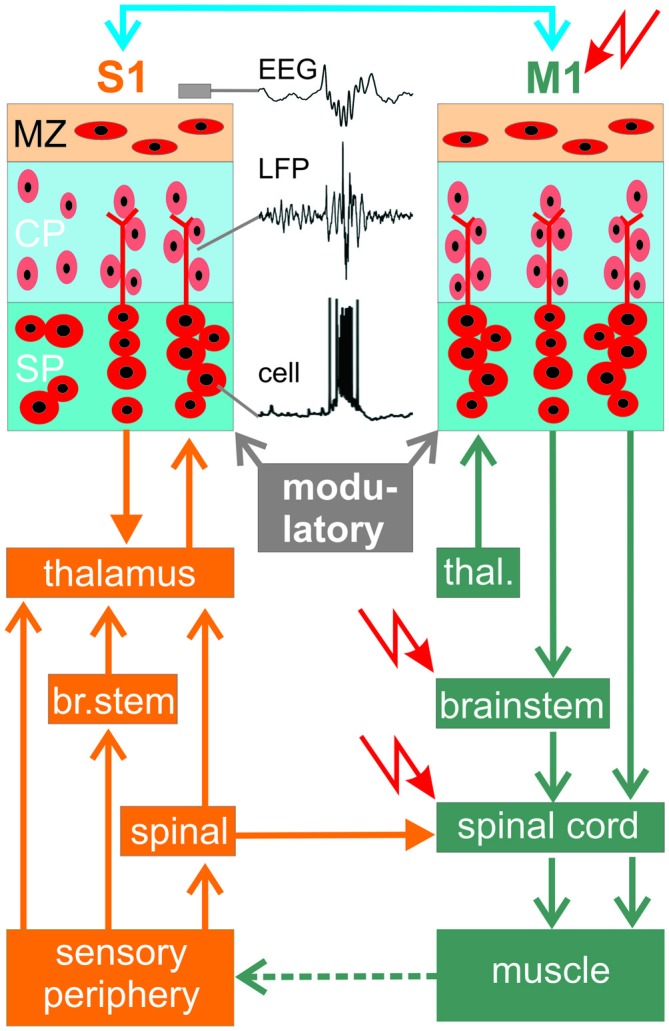
**Summary diagram showing connectivity between primary motor cortex (M1) and a primary sensory cortex area (here primary somatosensory cortex, S1) during early development.** Location of potential central pattern generators (CPGs) are indicated by 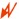. Traces in S1 illustrate spontaneous activity of a SP neuron, local field potential (LFP) activity in the CP and EEG recording on the cortical surface. Blue line at top indicates reciprocal corticocortical connections between S1 and M1.

Some early generated neocortical neurons may have the intrinsic capability to function as pacemakers. CRNs in newborn rodent cerebral cortex are functionally characterized by a prominent *I*_h_ (Kilb and Luhmann, 2000) and a low voltage-activated calcium channel (Kirmse et al., 2005). However, CRNs are insufficiently connected with other neocortical neurons to drive and synchronize early activity patterns (for review see Kirischuk et al., 2014; Luhmann et al., 2014). Further, in CRNs *I*_h_ does not contribute to spontaneous membrane potential shifts (Kilb and Luhmann, 2000). More likely, highly connected neurons with widespread axonal connectivity play a key role in synchronizing the activity of developing neocortical neurons. Such hub neurons, consisting of a subpopulation of GABAergic interneurons, have been found in developing hippocampal networks (Bonifazi et al., 2009; for review see Cossart, 2014). In developing cultured neocortical networks, synchronous oscillatory activity is driven by highly connected, large GABAergic preplate neurons, resembling SPNs (Voigt et al., 2001). Experimental data obtained from *in vitro* slice and *in vivo* preparations indicate that SPNs are capable to drive early synchronized network in newborn rodent cortex (Dupont et al., 2006; Hanganu et al., 2009; Tolner et al., 2012; Moore et al., 2014). SPNs may “amplify” their synaptic inputs and transmit the resulting oscillatory burst activity in a local columnar manner to the developing CP above (for review see Luhmann et al., 2009; Figure 3). Experiments in which the ablation of the SP by p75-immunotoxin abolished spontaneous spindle bursts and the columnar organization of the barrel cortex provided additional evidence for a causal role of SPNs in the generation of spontaneous oscillations and the neocortical architecture (Tolner et al., 2012).

However, SPNs are probably not the generators of the spontaneous neocortical activity, as demonstrated in diverse species and different cortical areas. For example, *in vivo* recordings in newborn rats have shown that spindle bursts in visual cortex are driven by spontaneous activity in the retina, the so-called retinal waves (Hanganu et al., 2006). Similar results have been obtained in the barrel cortex of newborn rats *in vivo*, where spontaneous spindle bursts and gamma oscillations are both reduced in their occurrence by about 50% when the electrical activity in the sensory periphery is silenced by injection of lidocaine into the contralateral whisker pad (Yang et al., 2009). Not only in newborn rats, but also in premature human neonates the spontaneous activity in sensory cortical areas is driven by activity of the sensory periphery (for review see Colonnese and Khazipov, 2012). SATs synchronize 87% of the spontaneous activity in the visual cortex and are eliminated following enucleation (Colonnese and Khazipov, 2010), providing further support that retinal waves drive the spontaneous activity in the very immature visual cortex. In mice, Zhang et al. (2011) used optogenetic techniques to directly manipulate retinal activity *in vivo* and demonstrated that the synchrony and precise temporal pattern of retinal activity is required for the development of eye-specific segregation and retinotopy. It is less clear whether activity in the sensory periphery also triggers early network activity in the auditory cortex. Inner hair cells in the cochlea of mice can generate spontaneous calcium action potentials as early as E17 (Marcotti et al., 2003). This early spontaneous activity of cochlear hair cells is triggered by a fluid secretion mechanism in adjacent glia-like support cells (Wang et al., 2015). The output of neighboring inner hair cells is synchronized via ATP-dependent signaling and triggers a theta-like burst of action potentials in auditory nerve fibers (Tritsch et al., 2007). *In vivo* recordings in the auditory brainstem suggest that inner hair cells act as pacemakers for spontaneous burst activity in more central auditory neurons before hearing onset (Tritsch et al., 2010). Taken together, these observations indicate that a substantial proportion of spontaneous activity observed in the developing sensory neocortex is caused by events in the sensory periphery or intermediary relay stations.

The activity from the sensory periphery (retina, cochlea) is transmitted to the cortex via direct connections to the thalamus (e.g., retino-geniculate in the visual system) or indirectly via the midbrain or brainstem (as in the auditory and somatosensory system, respectively; Figure 3). However, spontaneous activity arising from the sensory periphery lacks rapid oscillations, suggesting that the typical frequency of spindle bursts and gamma oscillations arise centrally. In the somatosensory system of newborn rodents, both activity patterns with their typical frequencies can be recorded already in the thalamus and synchronize a single thalamic barreloid with the corresponding neocortical barrel (Minlebaev et al., 2011; Yang et al., 2013). A corticothalamic feedback loop then modulates the thalamic network activity as demonstrated in newborn rats (Yang et al., 2013) and developing ferrets (Weliky, 1999).

Whereas the origin of spontaneous activity in the visual and auditory cortices may arise in the retina and sound-insensitive cochlea, respectively, the situation in the somatosensory system is more complex, because somatosensation (e.g., pain perception) is already present at birth (Mazzuca et al., 2011) and somatosensory activity is closely associated with motor activity. Newborn rats show during active (or REM) sleep, a behavioral state that predominates in early human infancy, hundreds of thousands of skeletal muscle twitches each day, which may be triggered by spontaneous activity in the spinal cord, brainstem or motor cortex (M1). The somatosensory feedback resulting from these spontaneous movements subsequently activates the somatosensory cortex (S1; Figure 3). In the somatosensory system of the neonatal rat *in vivo*, spatially confined spindle bursts in primary S1 are selectively triggered in a somatotopic manner by spontaneous muscle twitches (Khazipov et al., 2004). In human fetal development, spontaneous movements can be observed by ultrasound *in utero* already at the beginning of the second trimester, at a time point when the neocortical network has not been formed and is governed by a prominent SP (for review see Kostovic and Judas, 2010; Judaš et al., 2013). As an experimental model to study the development and the relationship between the sensory periphery and the cerebral cortex the whisker-to-barrel-cortex pathway in rodents proved to be most valuable, since here each whisker is represented in a somatotopic manner in the contralateral barrel cortex (for review see Feldmeyer et al., 2013). Rapid and asynchronous whisker movements in neonatal rats appear during active sleep and are related to cortical barrel-specific activity (Tiriac et al., 2012, 2014). These spontaneous whisker twitches appear before they are needed for behavior (active whisking during exploration) and are generated by central pattern generators (CPGs) located in the spinal cord, brainstem or primary M1 (red arrows in Figure 3).

Spontaneous activity is already present in the embryonic spinal cord and characterized by highly rhythmic episodes of motor neuron bursting activity that may drive muscle contractions. In the spinal cord of the mouse, spontaneous activity appears at E12.5, in the rat at E13 (for review see Moody and Bosma, 2005). During early stages, spontaneous activity depends on the excitatory action of GABA and glycine in embryonic spinal cord networks (for review see Sibilla and Ballerini, 2009). At this stage, developing spinal motor circuits are highly sensitive to the frequency and pattern of spontaneous activity, and drugs that alter this activity may cause developmental defects (Kastanenka and Landmesser, 2010). The excitatory action of both neurotransmitters is developmentally downregulated by a decrease in the expression levels of the neuron-specific potassium-chloride co-transporter type 2 (KCC2), reducing the intracellular chloride concentration and leading to a shift of equilibrium potential for chloride ions towards more negative values (Stil et al., 2011). Beside the spinal cord, the brainstem is another candidate to function as a potential CPG for spontaneous motor activity and movements during embryonic development (for review see Nakamura and Katakura, 1995; Figure 3). Facial motor neurons evoking rhythmic whisker movements have been detected in the brainstem and are part of a whisking CPG (Hattox et al., 2003). These whisking motor neurons receive synaptic inputs from the whisker representation in neocortical M1 (Hattox et al., 2002; Haiss and Schwarz, 2005; Cramer and Keller, 2006; Friedman et al., 2006), suggesting that more than one circuit and more than one brain region control the motor activity and the generation of movements. Interestingly, in this network electrical stimulation of even a single pyramidal cell in L5 of M1, or a single neuron in the facial nucleus, can elicit whisker movements (Brecht et al., 2004). Whereas activity in M1 activates brainstem reticular nuclei containing whisker premotor neurons mediating whisker protraction, activity in S1 excites premotor neurons in brainstem spinal nucleus trigeminalis interpolaris inducing a whisker retraction (Matyas et al., 2010; for review see Petersen, 2014).

However, these observations on the motor control of whisker movements have been obtained in adult rodents and it is less clear whether the M1 can elicit movements and subsequently an activation of the somatosensory system also in neonatal cortex. Anatomical studies in rodents have demonstrated the presence of corticomotor neuronal projections to the spinal cord before birth, but whether M1 drives muscle activity (e.g., in the form of twitches) in newborns is less clear. Direct electrophysiological proof for the functional role of this pathway comes from *in vivo* studies on newborn rats. Focal electrical stimulation of L5 in M1 at frequencies resembling the gamma oscillations (40 Hz) and spindle bursts (10 Hz) reliably elicited movements (An et al., 2014). About one quarter of the spontaneous gamma oscillations and spindle bursts in M1 triggered movements, which subsequently elicited gamma and spindle activity in S1 (An et al., 2014). This activity is most likely generated by the M1—brainstem/spinal cord—peripheral sensor—S1 pathway (Figure 3). A substantial proportion (~40%) of the spontaneous movements preceded the activity in M1 and blockade of the periphery by local lidocaine injection reduced the occurrence of gamma and spindle bursts by ~40% (An et al., 2014), indicating that motor-sensory interactions contribute to gamma and spindle burst activity in M1 of newborn rodents.

It has been shown in the mouse whisker system that the functional interaction between M1 and S1 does not depend on thalamic connections (Zagha et al., 2013), suggesting direct corticocortical connections between M1 and S1 (blue in Figure 3). Neuroanatomical tracing studies have demonstrated reciprocal connections between S1 and M1 (Aronoff et al., 2010; Mao et al., 2011). In preterm neonates simultaneous EEG and EMG measurements followed by Granger causality analysis have shown that M1 drives muscle activity, suggesting that corticomuscular communication in humans begins to develop during the late prenatal and neonatal stage (Kanazawa et al., 2014). As in developing neuronal networks of rodents, it is suggested that spontaneous activity in immature human cerebral cortex at some time point also depends on a high intracellular chloride concentration and an excitatory GABA action. SATs in human cerebral cortex show a decline by the time of normal birth. In age-matched fetal brain tissue, this decrease in SATs is correlated with a developmental up-regulation of KCC2 (Vanhatalo et al., 2005). Whether GABA may also have an inhibitory action at this early developmental stage, as recently suggested by Kirmse et al. (2015), remains to be studied in more detail in newborn awake animals by the use of non-invasive techniques addressing the influence of GABA on single cell firing and on network activity.

## Physiological Role of Spontaneous Activity and Pathological Consequences of Disturbances in Early Network Activity

An increasing amount of experimental data strongly indicates that spontaneous activity during very early ontogenetic stages is not simply an epiphenomenon of developing neuronal networks when they become electrically active, but rather plays important roles in various physiological processes in embryonic and early postnatal neocortical networks. One of the first developmental processes influenced by spontaneous neuronal activity is neurogenesis. Spontaneous retinal waves drive synchronized activity in the embryonic visual cortex of mice and modulate corticogenesis. Pharmacological inhibition of retinal waves increases neurogenesis and causes alterations in neocortical layering (Bonetti and Surace, 2010). Calcium waves in neuroproliferative radial glial cells in the VZ may be also directly involved in the regulation of neurogenesis, as: (i) the number of cells involved in them as well as the frequency and amplitude of calcium waves directly correlate to the proliferation in the VZ; and (ii) suppression of calcium waves drastically reduces proliferation in the VZ (Weissman et al., 2004). Further, even very basic morphogenic factors like sonic hedgehog are directly regulated by electrical activity (Belgacem and Borodinsky, 2015). Finally, the neurotransmitter identity of neurons can also be altered by electrical activity (Borodinsky et al., 2004). These examples indicate, that electrical activity strongly influences the generation and identity of neurons (for review see Kilb et al., 2011; Yamamoto and López-Bendito, 2012).

The overall number of neurons in the brain is determined by the number of generated neurons in relation to the number of dying neurons. Cell death is a fundamental physiological process in the developing brain (for review see Kuan et al., 2000) and is also modified by electrical activity. Already 6 h of spontaneous activity blockade induces a 2.5-fold increase in the number of neurons undergoing programmed cell death in neocortical cultures and organotypic slice cultures (Heck et al., 2008). Pharmacological interference with glutamatergic receptors has a similar impact on apoptosis, and blockade of GABA-A receptors causes a ~50% increase in cell death (Ikonomidou et al., 1999; Heck et al., 2008). Drugs that increase GABA-A receptor function (ethanol, antiepileptic drugs) also cause a prominent rise in apoptosis during early development (Ikonomidou et al., 2000; Ikonomidou and Turski, 2010). Lebedeva et al. (2015) recently demonstrated in rats that between P4 and P7, at the peak of ethanol-induced apoptosis, ethanol not only strongly suppressed spontaneous gamma and spindle bursts, but also sensory-evoked bursts and motor activity. These data indicate that any drug that modifies physiological activity patterns during early development may have an immediate impact on apoptosis.

The effect of GABA on apoptosis is cell type specific, as recent experiments revealed that inhibition of depolarizing GABAergic responses prevented apoptosis of CR neurons, while death rates of CP neurons were unaltered under this condition (Blanquie et al., 2016). Direct proof that the pattern of spontaneous activity controls the extent of programmed cell death comes from *in vitro* studies using multi-electrode arrays (MEAs) to record the activity in developing neocortical cultures. A reduction or delay in caspase-3 dependent apoptosis and an overall increase in neuronal survival could be observed in cultures showing high-frequency burst activity (Golbs et al., 2011), indicating that the physiological activity patterns observed in perinatal cerebral cortex *in vivo* have an impact on the control of cell survival vs. cell death. Brain-derived neurotrophic factor (BDNF) and activation of phosphatidylinositol 3-kinase (PI3K) and its downstream effector Akt are key downstream elements in the activity-dependent control of apoptosis (Wagner-Golbs and Luhmann, 2012). But also other survival-promoting pathways can be activated depending on the pattern of electrical activity or the site of calcium entry (for review see Hardingham and Bading, 2010; Bell and Hardingham, 2011). The ratio between cell death and cell survival determines brain volume. Studies in the developing human brain using EEG and quantitative magnetic resonance imaging have documented that an increased brain activity in the first postnatal days correlates with a faster growth of brain structures during subsequent months until term age. Particularly subcortical structures grew faster in babies with more SAT events (Benders et al., 2015).

Neuronal migration is another important process occurring in the cerebral cortex mostly during embryonic and early postnatal development (dependent on the species). Neuronal migration in the developing neocortex depends critically on the appropriate level of spontaneous activity (Bando et al., 2016). Spontaneous rhythmic intracellular calcium transients control neuronal migration (for review see Komuro and Kumada, 2005) and the two main cortical neurotransmitters, GABA and glutamate, have both a strong influence on migration (for review see Luhmann et al., 2015). The growth and differentiation of neuronal dendrites and axonal projections is also influenced by spontaneous activity (for review see Chen and Ghosh, 2005; Zheng and Poo, 2007; Yamamoto and López-Bendito, 2012). In neocortical cultures spontaneous synchronous network activity converts within a few minutes silent synapses to active synapses by incorporating AMPA receptors into the postsynaptic membrane (Voigt et al., 2005). Although a complex spatio-temporal pattern of transcription factors and intercellular communication mediated by constitutive secretion of transmitters or growth factors play an important role in the early development of thalamocortical axonal connections (for review see Molnár et al., 2003), spontaneous activity clearly contributes to the formation and refinement of topographic maps (for review see Hanganu-Opatz, 2010). A close interaction between spontaneous electrical activity and the expression of transcription factors has been demonstrated in the embryonic spinal cord, where blocking or slowing of bursting activity induces a downregulation of LIM homeodomain transcription factors (Hanson and Landmesser, 2004). In the mouse retinotectal system spontaneous retinal activity controls ephrinA-mediated responses and is required for the development of the retinotopic map and the elimination of exuberant retinal axons (Nicol et al., 2007).

Blocking spontaneous retinal activity in ferrets, which are born in a very immature state, during very early stages of development (P1–P10) caused a persistent disorganization of ocular dominance columns and a pronounced increase in receptive field size of neurons in primary visual cortex (Huberman et al., 2006). These data suggest that spontaneous retinal activity present before the onset of vision is required for the normal development of the columnar architecture (for review see Ackman and Crair, 2014). In newborn rat S1, spindle bursts and gamma oscillations originating from thalamic relay neurons synchronize local neocortical network in functional pre-columns (Minlebaev et al., 2011; Yang et al., 2013), indicating that these patterns of early activity might play an instructive role for the generation of the neocortical columnar architecture. This suggestion was corroborated by the observation that the attenuation of oscillatory activity after an ablation of SPNs affect the formation of barrels in the S1 (Tolner et al., 2012).

Neuronal activity, including spontaneous and sensory evoked activity, directly regulates axon myelination (Demerens et al., 1996; Barrera et al., 2013). This effect is partly mediated by an activity dependent differentiation of oligodendrocyte precursor cells via activation of adenosine receptors (Stevens et al., 2002). In addition, a vesicular, extrasynaptic release of the neurotransmitter glutamate from active axons can directly induce myelin formation in differentiated oligodendrocytes (Wake et al., 2015), via an NMDA receptor mediated and Fyn-kinase dependent release of translational repression (White and Krämer-Albers, 2014). Finally, in the early postnatal period it was recently shown that peripheral-driven neuronal activity also regulates vessel development and patterning in the developing rodent brain (Lacoste et al., 2014; Whiteus et al., 2014).

Further evidence for an important function of spontaneous activity in developing neocortical networks also comes from clinical studies. In humans, abnormal neocortical activity patterns recorded during perinatal stages predict the further development and the outcome in the following years. In extremely low gestational age infants, abnormalities in magneto-encephalography recorded somatosensory evoked magnetic fields at term age are associated with adverse neurodevelopment at 2 years of age (Rahkonen et al., 2013). Recently, Vanhatalo et al. (2005) have demonstrated that spontaneous bursts recorded with EEG in preterm infants exhibit scale-free properties and provide prognostic value of brain activity in the subsequent days (Iyer et al., 2015). A pilot study from the same group addressed the important question whether SATs in preterm babies are affected by drugs (phenobarbital, fentanyl, theophylline) that are routinely used in neonatal intensive care units. Although the visual EEG interpretation did not reveal any drug effects, advanced time-series analyses demonstrated that all drugs examined had an effect on spontaneous brain activity and may interfere with further development (Malk et al., 2014). In this context it is intriguing to note that both *in vitro* and *in vivo* experiments in rodents demonstrated that experimentally induced inflammation induces a rapid modification in the properties of spontaneous (spindle and gamma burst) activity, which subsequently causes an increase in programmed cell death (Nimmervoll et al., 2013). This study provides first evidence that altered neuronal activity may also contribute to the deleterious effects of inflammatory events in the immature human brain (Hagberg and Mallard, 2005).

## Current Challenges and Future Directions

Spontaneous activity in immature neocortical networks plays important roles during early and subsequent development. Although experimental animal studies and clinical data from preterm infants provided over the last decade a large amount of interesting and important results, a number of key questions remain to be addressed in the near future:

1. Does spontaneous neocortical activity fulfill a more general role in controlling activity-dependent processes or does a specific activity pattern have a distinct role during a certain stage of development in controlling a specific process?
2. What is the impact of disturbances in spontaneous activity caused by genetic, intrinsic (e.g., intrauterine infection, inflammation, hypoxia) or extrinsic factors (e.g., maternal medication or drug abuse) on different activity-dependent processes during corticogenesis? What are the long-term effects of these disturbances?
3. What are the characteristics of the normal spontaneous neocortical activity in the EEG recorded from full-term neonates and preterm infants? Which parameters can be extracted from the EEG to evaluate and quantify the spontaneous activity patterns? We need this information to evaluate the (patho-)physiological neuronal state of newborn and particular preterm babies during critical stages of their development.

## Author Contributions

HJL, AS, J-WY, VR-P, MCS, SK and WK wrote the article and generated the figures.

## Funding

We particularly thank our coworkers and the funding agencies, especially the Deutsche Forschungsgemeinschaft, for their continuous support.

## Conflict of Interest Statement

The authors declare that the research was conducted in the absence of any commercial or financial relationships that could be construed as a potential conflict of interest.
